# Divergent immune and extracellular matrix transcriptional programs underlie poor prognosis in early-stage lung adenocarcinoma

**DOI:** 10.3389/fimmu.2026.1762224

**Published:** 2026-04-16

**Authors:** Jyothi Thyagabhavan Mony

**Affiliations:** Independent researcher, San Diego, CA, United States

**Keywords:** early-stage lung adenocarcinoma, gene expression networks, immune-extracellular matrix interactions, overall survival, prognostic biomarkers, recurrence risk, TCGA-LUAD transcriptomic analysis, tumor microenvironment

## Abstract

**Background:**

Early-stage lung adenocarcinoma (LUAD) patients with similar pathological stage exhibit heterogeneous recurrence risk. Prior work suggested that intra-tumoral immune composition, particularly B cells, regulatory T cells, and macrophages stratify recurrence outcomes. These immune programs operate in the context of stromal and tumor-intrinsic transcriptomic programs.

**Objective:**

To identify coordinated gene expression programs linking immune, stromal and tumor-intrinsic processes to recurrence in early-stage (IA/IB) LUAD, and to assess their prognostic relevance in an independent cohort using overall survival.

**Methods:**

Gene expression data of 292 patients in NCBI GEO datasets was reanalyzed and unsupervised clusters were identified within the differentially expressed genes (DEGs) that associated with recurrence. Protective and risk-associated genes were identified in covariate-adjusted Cox models. GO enrichment was performed using DEGs. Pathway-level principal component scores were tested for association with overall survival in TCGA-LUAD early-stage, treatment-naive patients (n=90).

**Results:**

Modules enriched for B-cell and antibody responses, vascular homeostasis, and regulation of fibrotic remodeling were associated with reduced recurrence, whereas those enriched for epithelial organization and extracellular matrix (ECM) remodeling conferred increased risk. The contrast between ECM-driven recurrence risk and protective immune programs may reflect distinct biological states associated with recurrence timing, wherein changes in the ECM precede impaired lymphocyte responses in early-stage disease. In the TCGA cohort, immune-related programs associated with improved survival, whereas extracellular matrix (ECM) and epithelial differentiation/proliferation programs associated with worse survival.

**Significance:**

This study integrates immune, tumor, and stromal transcriptional programs to identify a prognostic dichotomy between ECM remodeling and adaptive immune-supportive states in early-stage LUAD. These coordinated tumor states and immune-matrix interactions offer mechanistic insight into recurrence and provide a framework for risk stratification.

## 1 Introduction

Lung cancer is one of the most common malignancies and the leading cause of cancer-related mortality in the United States and across the world (1, 2). Nearly 80% of all lung cancer diagnoses are accounted for by non-small cell lung cancer (NSCLC) and nearly half of these patients present with localized or locally advanced disease (3).

For patients with early-stage NSCLC, surgical resection remains the gold standard, offering the best chance for long-term survival. Following resection, prognosis is evaluated based on pathological stage, which reflects tumor size, nodal involvement, and metastasis. Even with complete resection and favorable pathological stage, a significant proportion of patients will experience disease recurrence (4). As such, recurrence serves as a biologically meaningful endpoint, reflecting residual micrometastatic disease, mutations and immune surveillance (5–8).

In the previous work, we showed that intra-tumoral immune composition is heterogeneous, and that the balance between Tregs, macrophages, and B cells can stratify recurrence risk in early-stage lung adenocarcinoma (9). The immune response operates in tandem with diverse processes such as remodeling of the extracellular matrix (ECM) and tumor-intrinsic reprogramming during tumor dissemination, immune evasion, and ultimately recurrence. As the neoadjuvant and adjuvant immunotherapies under clinical investigation (10, 11) begin to shape treatment strategies (4), there is a need to better understand the immune, stromal, and tumor-intrinsic programs that influence long-term recurrence risk and survival.

This follow-up study uses transcriptome-based approaches to identify distinct biological states in early-stage lung adenocarcinoma involving pathways driving differential immune engagement and recurrence trajectories. It further validates the gene expression pattern and processes associated with recurrence risk in a TCGA patient subset that did not receive adjuvant treatment using overall survival as a prognostic marker.

## 2 Methods

### 2.1 Microarray data acquisition and preprocessing

Raw microarray CEL files identified and used in the previous study (9) were retrieved from public datasets (GSE68465, GSE37745, GSE50081). These datasets had large numbers of rare stage IA/IB lung adenocarcinoma (LUAD) cases with long-term recurrence outcomes and recurrence-free survival data in patients not receiving adjuvant therapy. These datasets were used as they were readily amenable to the CIBERSORT deconvolution approach. The same dataset was revisited in this study for deeper analysis to test for consistency. Only samples with clearly annotated recurrence status were retained. CEL files were normalized using RMA via the affy R package and summarized with custom MBNI BrainArray CDFs (v25). Expression matrices were filtered for common genes and merged prior to batch correction.

### 2.2 Definition of recurrence groups

Recurrence kinetics were captured by stratifying the samples into six categories: Early Recurrence (<3 years), Late Recurrence (≥3 years), Short-Term Non-Recurrence (2–4 years), Durable Non-Recurrence (>4 years), Censored Short Follow-up (<2 years), and Healthy Lung. This classification enabled a more granular analysis than binary grouping used previously.

### 2.3 Batch correction, principal component analysis

Batch correction of merged expression matrices was performed with ComBat from the sva package. Two correction strategies were tested (1): correcting only for GPL96 and GPL570 platforms and (2) correction using a 13-level batch variable combining study ID with technical metadata as performed in the previous study. Principal component analysis (PCA) confirmed that the detailed batch correction used in the previous study effectively mitigated technical variation, allowing better resolution of biological structure. All analyses were performed with this detailed batch-corrected matrix.

### 2.4 Metadata integration and differential gene expression analysis

Sample metadata was cleaned and matched to expression data using standardized filenames. PCA coordinates were appended to metadata for visualization and adjustment. Matrices were aligned and assembled into ‘ExpressionSet’ objects using the Biobase package. The batch-corrected expression data was analyzed using the limma package. A relaxed filtering threshold (p < 0.05 and |log_2_FC| > 0.25) was applied to retain genes with potential biological relevance while minimizing false exclusions. This filtering strategy yielded a core gene set of 1,052 unique differentially expressed genes (DEGs) that was used for subsequent clustering and enrichment analyses. The processed microarray expression data is provided in **Supplementary Data Sheet 1**.

### 2.5 Unsupervised gene clustering and module eigengene calculation

DEGs were Z-score normalized and hierarchically clustered (Euclidean distance, complete linkage). Module assignments were derived using cutree() at k = 3, 9 and 18 to capture distinct biological resolutions. Module eigengenes (PC1 of each module) were computed using moduleEigengenes() from the WGCNA package (v1.72-1), resulting in a matrix of sample-level module activity profiles.

### 2.6 Gene-level survival analysis

CoxPH models showed statistically weak results at the eigengene-level due to functional heterogeneity within modules. Therefore, gene-level Cox models were used to identify survival-related transcripts. CoxPH was performed for each gene, adjusting for age, gender and tumor stage. Genes were labeled as protective (HR < 1) or risk-associated (HR > 1) for downstream enrichment and hazard visualization. For each module, the distribution of gene-level hazard directions (protective vs risk-associated) was used to identify dominant functional categories. Modules were interpreted by integrating both functional enrichment and the relative balance of protective versus risk-associated genes within the module.

### 2.7 Functional annotation of genes for visualization

Gene-level functional annotation was derived from Gene Ontology Biological Process (GO-BP) enrichment analysis performed using clusterProfiler::enrichGO() (12) with Benjamini-Hochberg adjustment (q < 0.05), using the 1,052 differentially expressed genes (DEGs) as the background. To reduce semantic redundancy among enriched GO terms, similarity-based clustering was performed using RRVGO (13), and representative terms were selected based on adjusted p-value ranking within each similarity cluster.

For downstream visualization and cross-module comparison (hazard plots and Sankey diagrams), enriched GO terms were consolidated into higher-order functional categories using predefined biological grouping rules based on shared mechanistic themes. For example, immunoglobulin production and plasma cell differentiation terms were grouped under “B cell/Immunoglobulin response”; chemotaxis and migration-related terms were grouped under “Chemokine/Migration” and retained as distinct from “Cytokine signaling”; and matrisome components including collagens, laminins, matrix metalloproteinases were grouped under “ECM/Adhesion/Remodeling.” When individual genes were associated with multiple enriched GO terms spanning different themes, assignment was based on module-level enrichment context and concordance of biological function across representative GO terms, and not on hazard direction. A complete mapping from genes to GO term to consolidated functional category and module assignment is provided in **Supplementary Table 1** to ensure transparency and reproducibility. Functional consolidation was applied solely for interpretative visualization and did not influence statistical testing or survival modeling.

### 2.8 Module label assignment and functional coherence

Genes within each module were grouped into curated higher-order functional categories derived from GO-BP enrichment and consolidated annotation. The number of genes per category was quantified, and categories represented by ≥4 genes were considered dominant functional components of the module.

Gene-GO term interaction networks were visualized using the cnetplot() function from the clusterProfiler package to assess functional coherence and topological clustering within each module. Final descriptive labels were assigned based on integrated biological patterns observed across constituent genes, enriched processes, and Gene-GO term interaction topology, rather than solely on the numerically largest category.

In the case of ME18, although cytokine-related categories were numerically prominent, network visualization revealed a coherent vascular-associated subnetwork linking BMP signaling, fibrinogen-associated genes, and vascular GO terms. Accordingly, the final module label reflects integrated network structure and biological coherence rather than raw gene count alone.

### 2.9 GO enrichment for protective vs risk genes

Protective and risk gene sets were separately enriched for official GO-BP terms using enrichGO() (parameters: p-adjust = BH, q-value < 0.05, minimum gene count = 3) and 1,052 DEGs as the background. Dot plots ranked by adjusted p-values were generated without *post hoc* grouping to preserve ontology structure. Two complementary statistical evaluations of module eigengenes were performed with respect to clinical recurrence patterns to prioritize co-expression modules for downstream biological and survival analysis. For survival validation in TCGA cohort, 184 genes spanning all 7 co-expression modules identified in the microarray analysis were selected to avoid restricting validation to only the four strongest recurrence-associated modules. This relaxed set included biologically relevant modules like ME1 enriched for cytotoxicity function. Pathway-level principal component scores were calculated for Cox proportional hazards modeling.

### 2.10 Sankey plot

To show the flow of gene functions through unsupervised clusters into the recurrence categories, a multi-layered Sankey input table was created by linking functional category to modules and to recurrence group, based on the dominant functions (≥4 genes/functional category) and the top pairwise Dunn contrast for the module. This visual framework summarized how gene-level biology flowed through co-expression modules into clinically distinct recurrence outcomes. Plots were generated using networkD3::sankeyNetwork() and exported as HTML.

### 2.11 TCGA-LUAD data acquisition, filtering criteria and stratification

RNA-seq expression and clinical metadata for lung adenocarcinoma (LUAD) patients were obtained from The Cancer Genome Atlas (TCGA) through the Genomic Data Commons (GDC) using the TCGAbiolinks R package. STAR-aligned raw counts were downloaded using the query parameters data.category = “Transcriptome Profiling”, data.type = “Gene Expression Quantification”, and workflow.type = “STAR - Counts”. Clinical metadata was retrieved using GDCquery_clinic(project = “TCGA-LUAD”, type = “clinical”). Consistent with the early-stage focus, data was filtered to include only pathologic Stage IA or IB disease. Histological purity was enforced by including only those patients with a primary diagnosis of “Adenocarcinoma, NOS”, excluding other adenocarcinoma subtypes such as mucinous, papillary, and bronchioloalveolar variants. The treatment-naive status was ensured by excluding samples from patients treated with chemotherapy or radiation therapy. Only patients with “no” recorded for both treatment types were retained.

For each patient, overall survival (OS) time was calculated as the number of days from diagnosis to death or last follow-up (days_to_death or days_to_last_follow_up) and converted to years. An event status was defined as 1 for death and 0 for censored observations. To approximate recurrence-based stratification used in the microarray analysis, patients were categorized into five surrogate recurrence groups based on their OS time and event status: Early_Recurrence: death within 3 years; Late_Recurrence: death after 3 years; ShortTerm_NonRecurrence: censored with follow-up between 2 and 4 years; Durable_NonRecurrence: censored with follow-up ≥4 years; Censored_ShortFollowup: censored with follow-up <2 years. This yielded a final cohort of 90 (non-duplicated) samples from patients with early-stage, untreated, lung adenocarcinoma and fully annotated follow-up information. The processed TCGA expression data is provided in Supplementary Data Sheet 2 and LUAD survival validation results are provided in **Supplementary Table 2**.

## 3 Results

### 3.1 Modular gene clustering reveals biologically distinct programs linked to recurrence patterns

**Figure 1:**
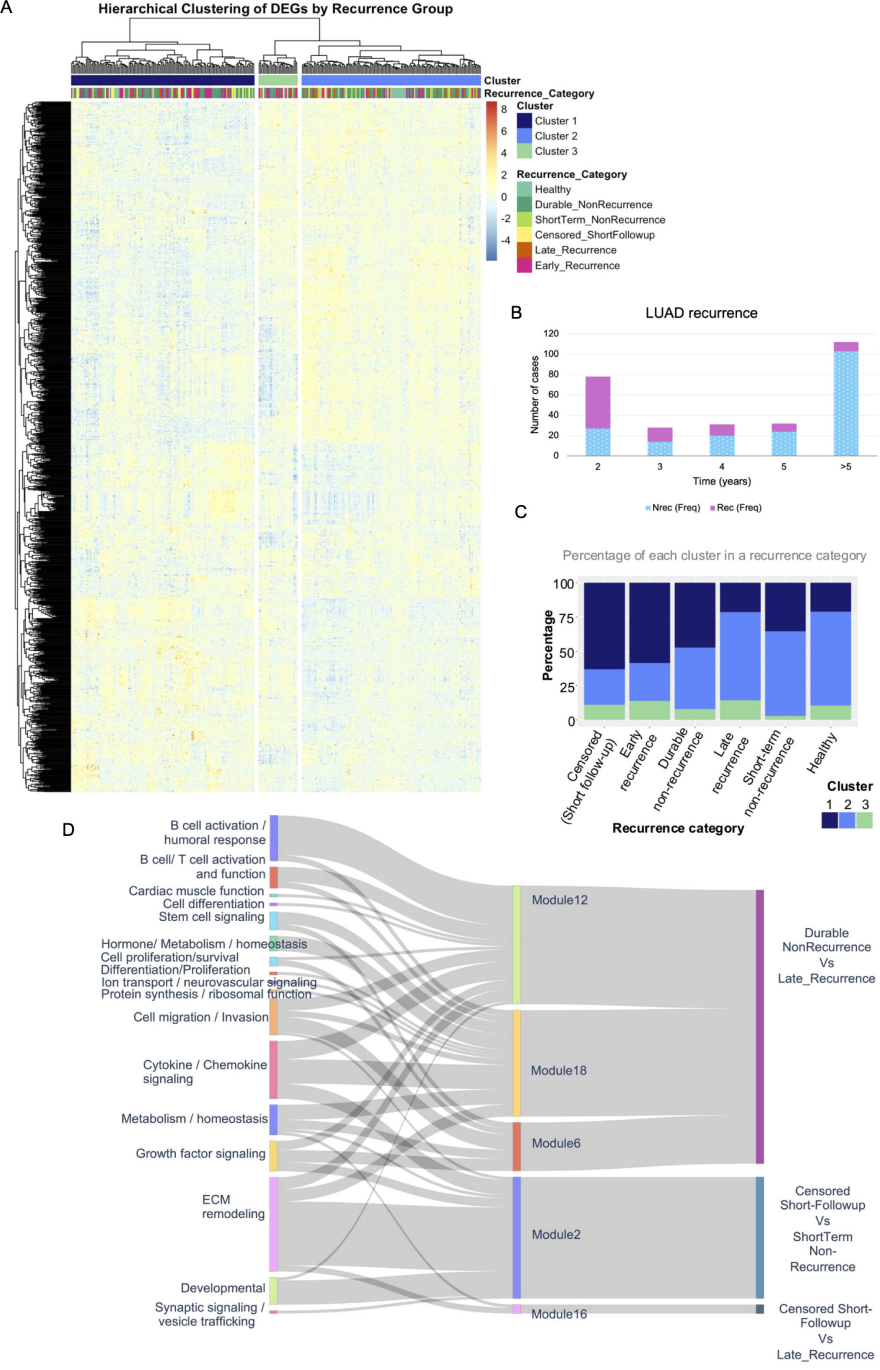
Transcriptome-wide structure of recurrence-associated gene expression. **(A)** Heatmap showing the hierarchical clustering of 1,052 differentially expressed genes scaled by Z-score across the LUAD samples in the columns. The heatmap was generated at k=3 to visualize the module-specific expression patterns across recurrence categories. Three sample clusters were identified using complete linkage and Euclidean distance. The clusters were visualized using colored column dendrogram segments (cutree k = 3). **(B)** Stacked barplot shows the distribution of recurrent and non-recurrent cases in the dataset **(C)** Stacked barplot showing the percentage of samples in each recurrence category assigned to each of the three expression-based clusters. Cluster 1 was the most prevalent among samples with poorer prognosis while Cluster 2 was enriched in healthy and recurrence groups with more favorable prognosis. Cluster 3 appeared less frequently but consistently across categories. **(D)** Sankey diagram linking functional annotation categories (left), co-expression modules (cutree k = 18, center), and the most significant recurrence group comparison per module (right). Functional categories were derived from gene-level GO enrichment and manual annotation. Only modules passing Spearman, Kruskal–Wallis, and Wilcoxon tests were included. Modules are color-coded by log2 hazard ratio (HR) from age-adjusted Cox proportional hazards analysis: red indicates higher recurrence risk (HR > 1), and blue indicates protective association (HR < 1). Line width of the links reflects the number of genes in each path from gene function through modules to recurrence. The recurrence group comparisons shown in the Sankey plot represent the most statistically significant pairwise differences in module eigengene expression. The organization of pairwise comparisons reflects underlying biological differences and was not annotated according to predefined recurrence timelines. Instead, this pattern emerged from the data-driven statistical analysis.

Transcriptional heterogeneity in early-stage LUAD was investigated using unsupervised hierarchical clustering of a filtered set of 1,052 differentially expressed genes identified from batch-corrected microarray data (**Figure 1A**). Most recurrences occured within 2 years in 51 out of 93 (∼55%) and late recurrences (>5 years) were rare (**Figure 1B**). A lower resolution (*k* = 3) was used to explore global co-expression structure (**Figure 1A**) and sample grouping (**Figure 1C**). A higher resolution (*k* = 18) was used to define refined co-expression modules used in downstream functional analysis (**Figure 1D**).

At *k* = 3, hierarchical clustering distinguished three major sample groups that exhibited subtle and biologically relevant differences across recurrence categories. Two of these clusters were of comparable size but differed in their recurrence composition. Cluster 2 was predominantly enriched in healthy lung and tumor samples from durable nonrecurrent cases, suggesting a protective transcriptional program. On the contrary, Cluster 1 was enriched in censored/short follow-up, early- and late recurrence cases suggesting a recurrence-risk associated transcriptional program. A third, smaller cluster enriched for Early Recurrence cases displayed a more divergent transcriptional profile. Crucially, the sample clustering suggested distinct transcription programs associated with the recurrence categories within early-stage LUAD.

At *k* = 18, four gene co-expression modules (ME2, ME6, ME12, ME18) demonstrated significant associations with recurrence status in both Kruskal-Wallis and Spearman correlation analyses. These modules were further tested in Dunn’s pairwise comparisons. The Sankey plot (**Figure 1D**) shows how module-level recurrence comparisons were linked with functional categories of the genes identified in unsupervised clusters. These recurrence-associated modules were functionally diverse: ME12 was enriched for immunoglobulin signaling and B cell-related genes suggestive of a protective humoral response; ME2 and ME6 captured ECM remodeling and Treg-related transcriptional programs, respectively, associated with higher recurrence risk; while ME18 displayed a mixed signature notably containing diverse genes including those regulating cell proliferation, differentiation, regulation of fibrosis, vascular and neuroimmune functions. The links weighted by the number of genes connected the gene function in the left through the unsupervised clusters to significant pairwise comparisons. The temporal pattern that emerges in pairwise comparisons is a spontaneous result of the statistical testing and highlights the distinct biological programs influencing recurrence outcomes over time. Particularly, the comparisons involving short follow-up cases were characterized by increased ECM changes (ME2). The Sankey diagram shows that transcriptional programs linked to recurrence involve overlapping yet distinct immune and tumor-intrinsic mechanisms, underscoring the complex biology underlying disease relapse.

### 3.2 Pro-tumorigenic and adaptive immune programs differ with recurrence timing

**Figure 2:**
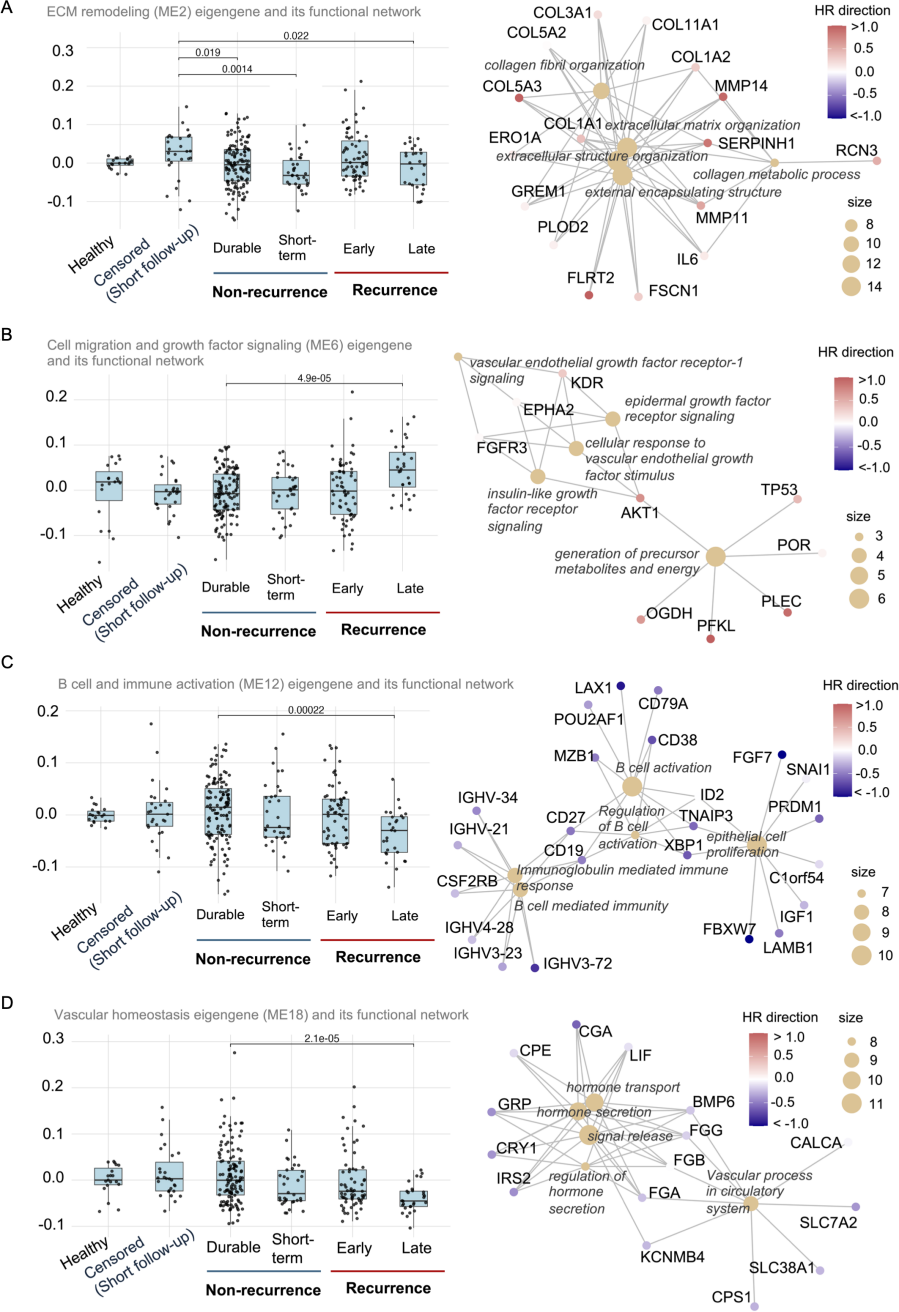
Module-level eigengene expression and functional network structure. Boxplots (left) show eigengene expression of selected modules across recurrence categories. These modules were prioritized based on significant Spearman, Kruskal-Wallis, and Wilcoxon tests. GO network plots (right) display enriched biological processes (nodes) and their associated genes (edges) within each module. Node size reflects the number of genes per GO term, and color indicates average log_2_ fold-change. These plots collectively illustrate the distinct biological programs linked to recurrence: **(A)** extracellular matrix remodeling, **(B)** cell migration and growth factor signaling, **(C)** B cell-mediated immune responses **(D)** vascular homeostasis.

To investigate whether biologically coherent transcriptional modules differed across recurrence groups, the expression patterns of the eigengene representing the first principal component of expression across all genes within the four most recurrence-associated gene modules (ME2, ME6, ME12, ME18) was examined (**Figure 2**).

ME2 (**Figure 2A**), enriched for extracellular matrix (ECM) remodeling pathways, had elevated expression in early recurrence samples and was significantly elevated in the censored short follow-up samples compared to Healthy and Durable Non-Recurrence groups. The network analysis revealed collagen organization and matrix remodeling involving COL1A2, MMP11, MMP14, and FN1 consistent with an invasive or stromal-activated transcriptional program. Interestingly, the cytokine IL6 is among the inflammatory genes in this module suggesting fibrosis and aberrant wound-healing responses.

ME6 (**Figure 2B**), characterized by classical growth factor signaling and metabolic genes driving tumor cell survival and proliferation displayed a progressive increase in eigengene expression across recurrence categories with the highest levels observed in Late Recurrence and Early Recurrence. Significantly higher levels of ME6 genes were detected in late recurrent samples compared to durable non-recurrence suggesting that distinct biological networks involving VEGFR, EPHA2 and IGF signaling may be contributing to angiogenesis, migration and survival gradually over time.

In contrast, ME12 (**Figure 2C**), enriched for B cell and immunoglobulin-related genes, displayed the highest eigengene expression in Healthy and Durable Non-Recurrence samples. Functional network analysis identified strong enrichment for plasma cell differentiation, immunoglobulin production, and humoral immune responses, including CD19, CD38, IGHV3-21, IGHV3-23, IGHV3-72, IGHV4-28, IGHV4-34, MZB1, POU2AF1, XBP1, and ID3. Moreover, this module was significantly downregulated in recurrent cases. Interestingly, this co-expression module also included CD200, the negative regulator of monocyte-macrophage axis. ME12 eigengene expression was significantly lower in late recurrence compared to Durable Non-Recurrence samples, highlighting a deficiency in adaptive immune responses marked by B cell-mediated immune surveillance.

Finally, the ME18 module (**Figure 2D**) captures a transcriptional program related to the vasculature. This module had a complex gene profile with temporal changes in eigengene expression that is lost over time with significant reduction in late recurrence cases compared to durable nonrecurrent cases. Functional enrichment revealed both protective and risk-associated signals. Intriguingly, several genes with hormonal and transporter functions in this module including GRP, CALCA, CALCB, CGA, CPE, KCNMB4, SLC38A1 and SLC7A2 have also been identified as having a neuroimmune function. This heterogeneous module also included matrix-associated fibrinogens (FGA, FGB, FGG). Importantly, the module included cytokines of bone morphogenetic protein (BMP) family, BMP2 and BMP6, and the downstream inhibitors of DNA binding 3 (ID3) suggesting a role for the regulation of fibrotic responses and vascular homeostasis.

Together, these module-specific eigengene patterns reinforce the notion that recurrence timing in early-stage LUAD is associated with distinct transcriptional programs, spanning immune activation and function, matrix remodeling, and altered lymphocyte function. The presence of both protective and risk-associated signatures within the same module further underscores the complexity of the immune-tumor interactions in solid tumors.

### 3.3 Gene-level recurrence risk reflects module-specific biological programs

**Figure 3:**
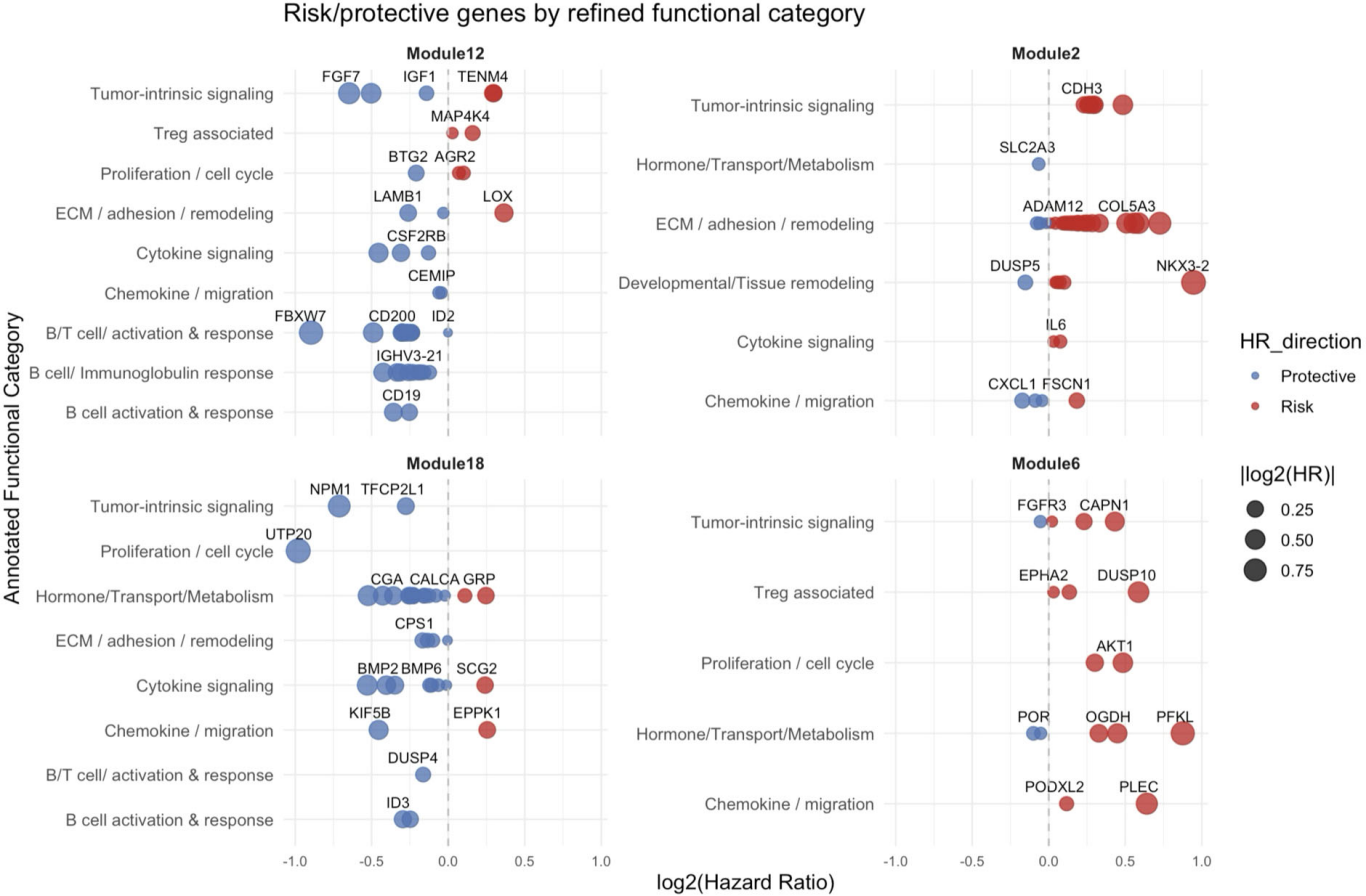
Gene-level hazard ratios grouped by functional annotation and module. Top genes from each recurrence-associated module (ME2, ME6, ME12, ME18) are plotted by log_2_ hazard ratio from age-adjusted Cox proportional hazards models. Genes are grouped along the y-axis by curated functional categories. Colors indicate direction of association (blue = protective, red = risk), and point size reflects |log_2_ (HR)| magnitude. Genes contributing recurrence risk including the ECM-related genes (e.g., *COL5A3, ADAM12*, *IL6*), and tumor-intrinsic drivers (e.g., *TP53*, *AKT1*) were predominantly enriched in ME2 and ME6 while the genes conferring protection (e.g., *IGHV3-72*, *CD19*), macrophage checkpoint (*CD200*) can be found predominantly in ME12 and ME18.

Cox proportional hazards modeling was performed for each gene in the recurrence-associated modules, adjusting for age, gender, and tumor stage to identify the genes that contribute most strongly to recurrence risk. **Figure 3** displays top-ranked genes from four key modules (ME2, ME6, ME12, ME18), grouped by curated functional categories and plotted by log_2_ hazard ratio (HR).

The distribution of both protective (log_2_ HR < 0) and risk-associated (log_2_ HR > 0) genes across annotated functional categories within each module reveals distinct trends. The protective responses included B cell-mediated immune functions within ME12 and diverse functions in ME18 involving hormone and transporter genes linked to neuroimmune communication and vascular homeostasis, along with BMP-signaling pathways with known anti-fibrotic potential. The risk for recurrence was associated with ECM remodeling within ME2. Collectively, these plots emphasize that recurrence risk in early-stage LUAD is shaped by the interplay of pro-tumor stromal remodeling and immune-based protective signals.

### 3.4 Top functional pathways linked to recurrence vary by module and risk direction

**Figure 4:**
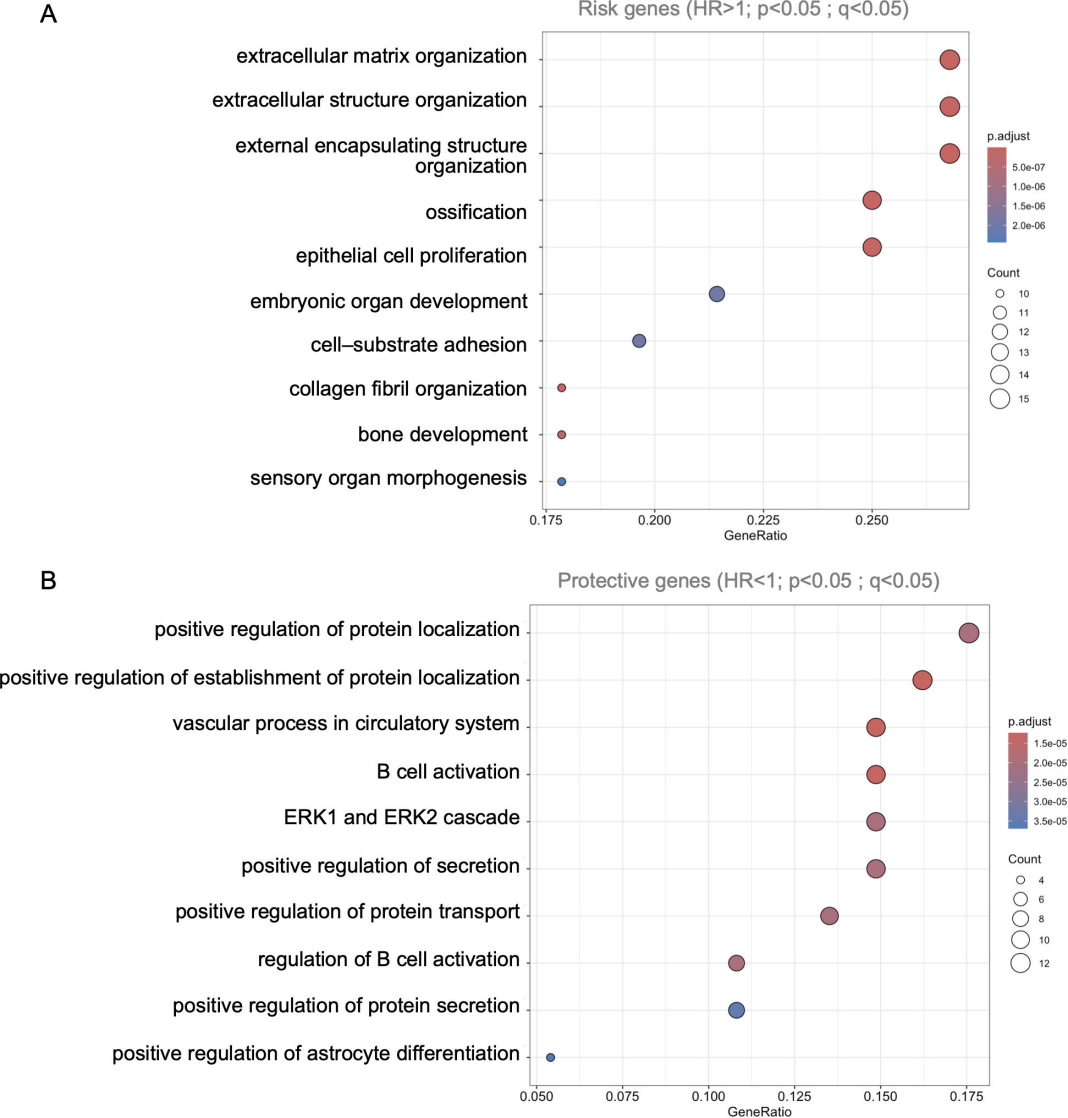
Functional enrichment of protective and risk-associated genes based on survival modeling. Gene Ontology Biological Process (GO-BP) enrichment was performed separately for genes associated with **(A)** reduced recurrence risk (HR < 1) and **(B)** increased recurrence risk (HR > 1), using age-adjusted Cox proportional hazards models. Dot size reflects the number of genes per term, and color indicates the adjusted p-value. The protective genes were enriched in immune-related functions, vascular processes, and secretory pathways while risk-associated genes were strongly enriched in extracellular matrix organization, epithelial proliferation, and developmental processes.

GO-BP enrichment was performed to identify biological processes most strongly linked to recurrence risk (**Figure 4**). The genes from the four significant modules (ME2, ME6, ME12, ME18) were stratified separately into protective (HR < 1, **Figure 4A**) and risk-associated (HR > 1, **Figure 4B**) subsets. To focus on the most prognostically relevant biology, the top three enriched pathways (ranked by gene count) were selected from each module.

This targeted approach revealed distinct functional themes associated with recurrence timing. Protective gene signatures were dominated by immune-related processes, particularly cytokine signaling, B cell activation, and adaptive immune responses, especially within ME12 and ME18. In contrast, risk-associated pathways included extracellular matrix remodeling, epithelial proliferation, development and differentiation related programs, with the strongest enrichments observed in ME2 and ME6. These findings suggest that recurrence is driven by programs such as stromal remodeling and proliferative signaling, while non-recurrence is associated with robust adaptive immune response driven by B cells.

### 3.5 Survival associations of gene ontology pathways in TCGA-LUAD

**Figure 5:**
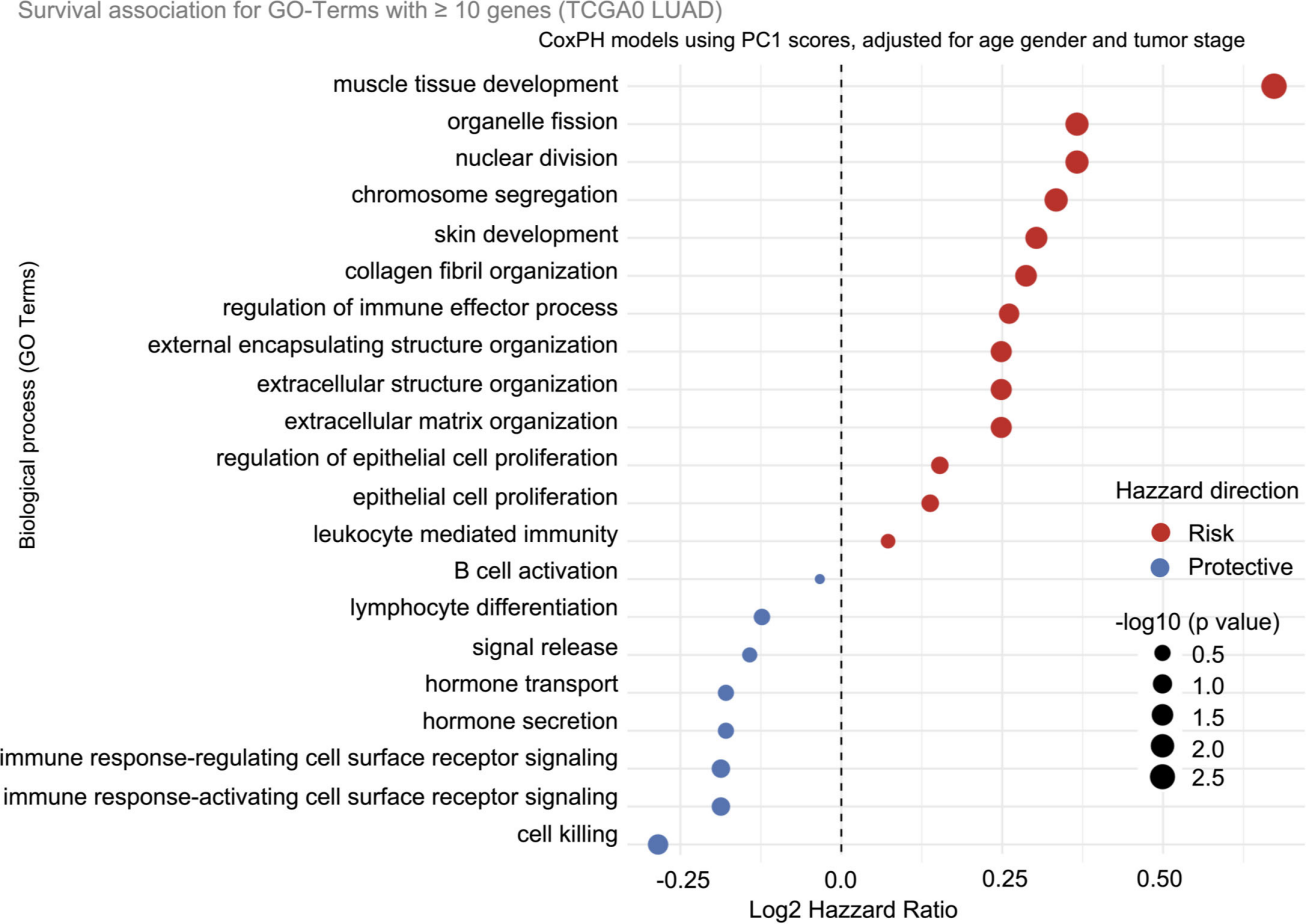
GO-term PC1 hazard ratios from TCGA-LUAD, adjusted for age, gender, and tumor stage. Each dot represents the association between a biological process (GO term) and overall survival, quantified as a hazard ratio (log_2_ scale) derived from Cox proportional hazards modeling using the first principal component (PC1) of gene expression for each term. Several immune-related GO terms including Cytotoxicity, B cell activation, immunoglobulin-mediated immune response, and adaptive immune signaling were associated with favorable prognosis, while terms associated with tumor differentiation, proliferation, development, and importantly the acellular extracellular matrix were associated with increased hazard.

The prognostic relevance of biological processes identified in the microarray-based recurrence analysis was further tested for association with overall survival (OS) in the TCGA-LUAD cohort (n = 90). For each GO Biological Process, a principal component score (PC1) was computed from the expression of member genes across tumor samples. These PC1 scores were then analyzed using Cox proportional hazards (CoxPH) models adjusted for age, gender, and tumor stage (**Figure 5**). The results demonstrated consistent directional associations between biological processes and overall survival. Nominal statistical significance (p < 0.05) was observed for 9 of 21 tested biological processes, with log2(HR) values ranging from -0.28 to 0.68, corresponding to approximately an 18% reduction in hazard for protective processes and up to a 60% increase for risk-associated processes.

Risk-associated processes involving epithelial proliferation, dedifferentiation, developmental pathways, and extracellular matrix remodeling had the largest positive hazard ratios and included several terms that remained significant after FDR correction. In contrast, multiple immune-related processes including cytotoxicity, B cell activation, immunoglobulin-mediated immune response, and immune response activating receptor signaling, exhibited consistently negative log_2_ hazard ratios, indicating a modest protective directional bias.

To evaluate this directional separation, GO terms were grouped into broader immune and non-immune categories, and the distribution of log_2_ hazard ratios was compared using a Wilcoxon rank-sum test. Immune-associated processes demonstrated a significant leftward shift toward protection relative to non-immune programs (W = 17, p = 0.0185; **Supplementary Figure 1**).

Non-immune programs, predominantly tumor-intrinsic and stromal pathways, exhibited larger positive hazard ratios and included several processes that remained significant after multiple testing correction (p < 0.05; FDR ≤ 0.1). These included processes related to extracellular matrix organization and epithelial structural remodeling. In contrast, immune-associated pathways consistently showed hazard ratios < 1, indicating protection, but with comparatively smaller effect sizes.

Notably, processes related to extracellular matrix organization and epithelial proliferation signaling were reproducibly associated with increased recurrence and mortality risk across both the microarray and TCGA RNA-seq datasets.

## 4 Discussion

Understanding the biological processes involved in disease progression in early-stage lung cancer using recurrence-free survival (RFS) along with overall survival (OS) offers distinct advantages. Recurrence reflects the failure of curative-intent therapy (surgical resection) to eliminate micrometastatic disease active at the time of resection and can be linked to tumor biology and immune evasion mechanisms. Our previous study indicated that recurrent tumors harbor an intra-tumoral immune composition characterized by increased Tregs and macrophages and reduced plasma cells and effector CD4^+^ T cells, consistent with impaired early immune surveillance (9). While recurrence points to failures in immediate tumor clearance, OS reflects the cumulative influence of tumor burden and persistent immune competence. OS can be influenced by factors such as age-related comorbidities, treatment complications, access to care, and disease management strategies. These factors can obscure biologically relevant signals, especially in early-stage disease where patients may live for years despite recurrence or other health challenges. In this study, recurrence-based analysis of the microarray dataset identified prognostic immune and matrix-remodeling programs. These findings were independently validated in the TCGA-LUAD RNA-seq cohort using overall survival as the clinical endpoint. Although recurrence and overall survival represent distinct clinical endpoints, the consistent directional associations across platforms suggest shared underlying biological programs.

Early-stage lung adenocarcinoma patients with similar TNM stage may differ in the risk of recurrence based on immune-tumor interactions. Whole-tumor transcriptomic analyses can identify molecular drivers of post-resection failure and immune dysfunction. These insights may aid risk stratification and follow-up strategies. To assess translational relevance, recurrence-associated genes identified in the microarray dataset were evaluated for their association with overall survival in the TCGA-LUAD cohort. This approach allowed a focused evaluation of immune and stromal pathways across platforms and clinical outcomes.

The results were consistent with our previous study and confirmed a protective role for adaptive immune responses involving B cells. Cytolytic immune processes generally associated with NK, CD8+ and γδ T cell populations were enriched in Module 1. However, this module did not have a significant association with recurrence. The pathway-level survival modeling in the smaller TCGA-LUAD cohort revealed that immune processes had modest protective association with overall survival. In our previous study, early-stage lung cancer patients with strong B cell/plasma cell responses could be further stratified based on concomitant myeloid activation, which was associated with increased recurrence risk (9). Consistent with the importance of myeloid regulation in this context, CD200 (OX-2), an immune checkpoint ligand that engages the inhibitory receptor CD200R1 expressed predominantly on myeloid cells, was associated with reduced recurrence risk. This result is consistent with the protective association of CD200 in lung cancer (14). Notably, CD200 expression clustered with genes involved in B cell activation and adaptive immune responses within Module 12, suggesting that CD200-mediated modulation of myeloid cell activity may occur within protective B cell-rich tumor microenvironments in early-stage disease. However, this protective effect of CD200 is likely context dependent. CD200 signaling can promote the differentiation of regulatory T cells and expansion of myeloid-derived suppressor cells (15, 16). The blockade of CD200 signaling has demonstrated therapeutic benefit in preclinical models of cancers (16, 17). The CD200 blocking antibody samalizumab was shown to enhance phagocytosis of tumor cells by PBMC (17). Conversely, CD200 signaling also plays a critical role in maintaining myeloid homeostasis, by limiting myelopoiesis in the bone marrow and restrains myeloid/macrophage responses during inflammation (18–20). In the context of early-stage LUAD, the favorable association of CD200 expression may therefore reflect regulation of intratumoral myeloid responses. Notably, in the absence of regulation by CD200, alveolar macrophages can produce increased levels of IL6 (20). IL6 signaling has been implicated in driving fibrosis during injury and recurrent inflammation (21, 22).

Stromal remodeling emerged as an independent axis of recurrence risk. Gene module (ME2) was enriched for collagen organization, matrix disassembly, and extracellular structure organization processes and included the cytokine IL6, multiple collagens and matrix metalloproteins (MMPs). IL6 trans-signaling by binding soluble IL6R can induce collagen expression in healthy dermal fibroblasts by augmenting Transforming Growth Factor-β pathway (23). IL6 produced by M2-macrophages can activate fibroblasts and induce collagen expression (24). IL6/STAT3 signaling can also increase MMP (1, 3 and 13) expression in tumor cells driving tumor invasion (25, 26). This dual role of IL-6 in collagen deposition and matrix degradation reflects a dynamic, fibrosis-like stromal remodeling process characteristic of tumor desmoplasia (27). Consistent with aberrant stromal physiology, MMP11 and MMP14 co-clustered with IL6 and collagen-associated genes within the recurrence-associated ME2 module, suggesting that this module reflects a coordinated, fibrosis-like remodeling program rather than isolated matrix degradation. MMP11 has been reported as a potential therapeutic target in lung adenocarcinoma, where its depletion and antibody-mediated inhibition were found to impair tumor cell growth, migration, and invasion *in vitro* and in xenograft models (28).

MMP activity can facilitate immune cell infiltration through matrix degradation and produce bioactive peptides that can modulate the immune response. If MMP-mediated proteolysis primarily promoted immune activation, it would be reasonable to expect these genes to be co-expressed with adaptive immune signatures and associate with protective effect. Prior studies have reported associations between MMP14 expression and immune infiltration (29). In the present analysis, however, MMP11 and MMP14 co-clustered with IL6, collagens, and additional fibrosis-associated genes within ME2, a recurrence-associated module enriched for extracellular matrix remodeling and aberrant wound-healing-like responses. This module demonstrated coherent risk-associated hazard ratios across multiple structurally and functionally related genes, including MMP14 (log_2_HR ~0.55) and collagen fibril organization programs in TCGA validation.

In contrast, lysyl oxidase (LOX) involved in collagen crosslinking and fibrosis clustered within the immune-enriched ME12 module, which was dominantly protective at the program level. Although LOX exhibited an individual risk-associated hazard ratio (log_2_HR ~0.35), it represented a minority stromal component within an otherwise B cell-dominated transcriptional program characterized by immunoglobulin genes and adaptive immune signaling. This distinction underscores why biological interpretation was grounded in module-level and pathway enrichment rather than isolated single-gene behavior. By comparison, MMP11 and MMP14 co-clustered within a transcriptionally coherent extracellular matrix remodeling program. These genes had stronger and directionally aligned risk-associated hazard ratios, and ECM-related GO terms were independently associated with increased hazard in the TCGA survival validation.

Collectively, these findings support the interpretation of a dysregulated stromal remodeling axis distinct from adaptive immune programs. ECM-rich tumors may represent fibrosis-like structural remodeling that constrains lymphocyte infiltration. In contrast, B cell-enriched tumors exhibit features of permissive immune surveillance and protective immunity.

The ECM can also act as stores for cytokines and growth factors that modulate tumor cell survival, proliferation and differentiation. In the TCGA cohort, GO terms indicative of epithelial structural programs related to intermediate filament organization, cytoskeletal reorganization and keratinocyte differentiation was significantly associated with increased risk for survival. Collectively, while several ECM remodeling processes associated with increased risk of recurrence in the microarray dataset, epithelial cell proliferation and a less differentiated tumor phenotype were linked to poor overall survival in the TCGA dataset. Thus, these results reveal distinct patterns in tumor-intrinsic and immune-mediated biological programs in early-stage lung adenocarcinoma.

In contrast to the ECM-associated risk programs, ME18 module exhibited a distinct transcriptional profile characterized by genes involved in vascular regulation, neuroendocrine signaling, and control of fibrotic responses. It is defined by a transcriptional signature that integrates neuroendocrine regulators (CGA, CPE), neuropeptide vasodilators (CALCA, CALCB), ion channel regulators (KCNMB4), amino acid transporters involved in endothelial metabolism (SLC7A2, SLC38A1). Collectively, these co-expressed genes may represent a coordinated neuroimmune-vascular interface within the tumor microenvironment. Notably, vascular-associated transcriptional programs within this module demonstrated protective characteristics, suggesting that stromal and vascular states that support tissue organization and immune accessibility. Because the vasculature serves as the primary gateway for lymphocyte trafficking, these findings support the concept that vascular integrity and signaling programs influence immune composition and infiltration in early-stage lung adenocarcinoma.

Developmental cytokines from the TGF-β superfamily, including BMP2 and BMP6, also clustered with the vasculature-associated genes within Module ME18. These factors may contribute to the protective characteristics of this module by regulating fibrosis and supporting vascular homeostasis. Mechanistically, BMP signaling is known to counteract TGF-β1-driven profibrotic pathways, including suppression of Smad2/3 activation (30), myofibroblast differentiation (31), and collagen production (32) across multiple tissue contexts. Experimental studies have demonstrated that BMP ligands can reduce collagen deposition and fibrotic remodeling (31, 33), while loss of BMP signaling enhances fibroblast proliferation and collagen expression (32). Consistent with these antifibrotic effects, BMP pathway activation has also been associated with improved resolution of lung fibrosis in preclinical models. In connection with the vasculature, BMP2 and BMP6 can induce the expression of angiogenic regulators such as ID family transcription factors (34) and promote endothelial branching and vascular density (35).

Collectively, these observations suggest that the BMP-enriched transcriptional program within Module ME18 may reflect a stromal state characterized by controlled matrix remodeling and preserved vascular integrity. Such a microenvironment could facilitate immune cell trafficking and surveillance, consistent with the protective association of this module with reduced recurrence. These findings are consistent with the concept of vascular normalization proposed by Jain RK, in which restoration of vascular structure and function improves perfusion and immune infiltration (36, 37).

While RNA expression might not fully account for post-transcriptional regulation and protein-level function, many biological processes (e.g., cytokine production, immune infiltration, ECM remodeling, lymphocyte activation) are transcription driven. Thus, transcriptome-wide analysis provides a practical and scalable framework for identifying broad biologically meaningful patterns such as those identified in this study. Importantly, the transcriptional programs identified here were independently evaluated at the gene level and further validated in an external TCGA cohort using pathway-level survival modeling. These orthogonal analyses support the robustness of the identified immune and ECM-associated signatures.

The coordinated enrichment of MMP14, fibrillar collagens, and IL6 within ME2 suggests a spatially organized desmoplastic niche. A specific hypothesis emerging from this analysis is that MMP14-expressing macrophage subsets localize preferentially within collagen-rich tumor regions, where they contribute to extracellular matrix remodeling and IL6-associated fibrotic signaling. This may reinforce a desmoplastic microenvironment that restricts adaptive immune infiltration, including B cell responses, thereby promoting recurrence. Within this framework, the CD200 expression by B cells and endothelial cells could counterbalance by restraining macrophage activation and limiting excessive IL6-driven fibrotic responses. Such context-dependent modulation of myeloid activity could contribute to maintaining a microenvironment that permits adaptive immune infiltration in early-stage tumors.

A limitation of the TCGA validation cohort is the relatively small number of unique, early-stage, untreated LUAD cases (n = 90) that met the stringent inclusion criteria. The TCGA cohort contained a limited number of observed events (n = 20), with a substantial proportion of censored short-follow-up cases, which may reduce statistical power. While tumor-intrinsic programs retained strong statistical associations, the immune and vascular-related pathways had hazard ratios in protective direction but with weaker statistical strength. This pattern likely reflects limited sample size rather than biological inconsistency. Despite the limited number of observed events in the TCGA cohort, multiple tumor-intrinsic ECM, cytoskeletal, and epithelial differentiation/proliferation programs remained significant at FDR ≤ 0.1, with consistent hazard ratios >1.25, indicating that these pathways represent important prognostic axes in early-stage LUAD.

Future validation in larger, multi-center, early-stage cohorts with annotated recurrence data will be important to confirm the robustness of these immune-ECM associations. Other publicly available resources such as the International Cancer Genome Consortium (ICGC) and additional RNA-seq datasets with long-term recurrence annotation may provide opportunities for further validation of these transcriptomic programs in independent populations.

## 5 Conclusion

This study identifies distinct biological mechanisms and gene programs linked to recurrence and survival risk in early-stage lung adenocarcinoma. Specifically, the analysis reveals (1): an immune axis characterized by protective B cell responses consistent with our previous study (2); a dichotomy between immune-mediated protection and extracellular matrix remodeling that shows temporal association with recurrence; and (3) a favorable niche defined by regulated tumor stroma, vascular signaling, and epithelial maintenance.

Collectively, the findings suggest that a balance between stromal remodeling programs and immune activation states determines recurrence risk in early-stage LUAD patients undergoing surgical resection. The protective module enriched for vascular and BMP signaling genes may reflect a stromal state that counterbalances TGFβ-driven pro-fibrotic responses within tumors. BMP signaling has been reported to antagonize TGF-β-mediated fibroblast activation and excessive matrix deposition in fibrotic contexts. In contrast, the recurrence-associated ECM module demonstrated coordinated upregulation of collagens, IL6, and matrix remodeling enzymes consistent with dysregulated desmoplasia.

These observations raise the possibility that restoring stromal balance either by attenuating IL6/TGF-β-driven fibrosis or promoting BMP-associated homeostatic signaling may represent candidates for combinatorial strategy to enhance immune permissiveness in early-stage LUAD.

Interestingly, the cross-sectional comparisons suggest that ECM-driven stromal remodeling programs are more prominent in early recurrence groups, whereas reduced vascular homeostasis and BMP- signaling are more evident in late recurrence cases. Although these observations do not establish longitudinal evolution, they raise the possibility that distinct microenvironmental states may characterize different recurrence kinetics. If validated in larger cohorts, this could suggest distinct biological vulnerabilities, with early recurrence and late recurrence. These findings suggest that these combinatorial therapeutic strategies may benefit from biological stratification rather than uniform application across all early-stage LUAD patients. The module-derived scores reflecting ECM remodeling, immune activation, or vascular/BMP-associated signaling, as well as context-dependent immune regulators such as CD200, warrant prospective evaluation as candidate biomarkers for risk-adapted therapeutic approaches.

## Data Availability

Publicly available datasets were analyzed in this study. These datasets can be found here: The NCBI Gene Expression Omnibus (GEO) under accession numbers GSE68465: https://www.ncbi.nlm.nih.gov/geo/query/acc.cgi?acc=GSE68465; GSE37745: https://www.ncbi.nlm.nih.gov/geo/query/acc.cgi?acc=GSE37745; GSE50081: https://www.ncbi.nlm.nih.gov/geo/query/acc.cgi?acc=GSE50081. TCGA-LUAD RNA-seq data and associated clinical metadata were obtained from The Cancer Genome Atlas (TCGA) via the Genomic Data Commons (GDC) portal (https://portal.gdc.cancer.gov/). All processed expression matrices, module assignments, gene-level annotations, GO enrichment results, and statistical analysis outputs supporting the conclusions of this article are provided in the **Supplementary Data Files**. The R scripts used for preprocessing, clustering, enrichment analysis, survival modeling, and figure generation are available from the author upon reasonable request.

## Supplementary material

Supplementary Figure S1 Distribution of GO-term hazard ratios by biological category. Log2 hazard ratios derived from PC1-based Cox proportional hazards models in the TCGA-LUAD cohort were stratified into immune-related and non-immune biological processes. Immune-associated GO terms demonstrated a significant protective association compared to non-immune terms (Wilcoxon rank-sum test).

## References

[1] Siegel RL Kratzer TB Giaquinto AN Sung H Jemal A . Cancer statistics, 2025. CA Cancer J Clin. (2025) 75:10–45. doi: 10.3322/caac.21871. PMID: 39817679 PMC11745215

[2] Bray F Laversanne M Sung H Ferlay J Siegel RL Soerjomataram I . Global cancer statistics 2022: GLOBOCAN estimates of incidence and mortality worldwide for 36 cancers in 185 countries. CA Cancer J Clin. (2024) 74:229–63. doi: 10.3322/caac.21834. PMID: 38572751

[3] Howlader N Forjaz G Mooradian MJ Meza R Kong CY Cronin KA . The effect of advances in lung-cancer treatment on population mortality. N Engl J Med. (2020) 383:640–9. doi: 10.1056/NEJMoa1916623. PMID: 32786189 PMC8577315

[4] Fick CN Dunne EG Vanstraelen S Toumbacaris N Tan KS Rocco G . High-risk features associated with recurrence in stage I lung adenocarcinoma. J Thorac Cardiovasc Surg. (2025) 169:436–444.e6. doi: 10.1016/j.jtcvs.2024.05.009. PMID: 38788834 PMC11582076

[5] Aliperti LA Predina JD Vachani A Singhal S . Local and systemic recurrence is the achilles heel of cancer surgery. Ann Surg Oncol. (2011) 18:603–7. doi: 10.1245/s10434-010-1442-0. PMID: 21161729 PMC11156256

[6] Remark R Becker C Gomez JE Damotte D Dieu-Nosjean MC Sautès-Fridman C . The non-small cell lung cancer immune contexture: A major determinant of tumor characteristics and patient outcome. Am J Respir Crit Care Med. (2015) 191:377–90. doi: 10.1164/rccm.201409-1671PP. PMID: 25369536 PMC5447326

[7] Yan Q Li S He L Chen N . Prognostic implications of tumor-infiltrating lymphocytes in non-small cell lung cancer: a systematic review and meta-analysis. Front Immunol. (2024) 15:1476365. doi: 10.3389/fimmu.2024.1476365. PMID: 39372398 PMC11449740

[8] Zhou C Jing Z Liu W Ma Z Liu S Fang Y . Prognosis of recurrence after complete resection in early-stage lung adenocarcinoma based on molecular alterations: a systematic review and meta-analysis. Sci Rep. (2023) 13:18710. doi: 10.1038/s41598-023-42851-2. PMID: 37907475 PMC10618289

[9] Mony JT Schuchert MJ . Prognostic implications of heterogeneity in intra-tumoral immune composition for recurrence in early stage lung cancer. Front Immunol. (2018) 9:2298. doi: 10.3389/fimmu.2018.02298. PMID: 30374348 PMC6196259

[10] Wakelee H Liberman M Kato T Tsuboi M Lee S-H Gao S . Perioperative pembrolizumab for early-stage non–small-cell lung cancer. N Engl J Med. (2023) 389:491–503. doi: 10.1056/nejmoa2302983. PMID: 37272513 PMC11074923

[11] Aguado C Jiménez Maestre UJ Mielgo-Rubio X . Neoadjuvant immunotherapy in non-small-cell lung cancer: times are changing—and fast. World J Clin Oncol. (2022) 13:758–61. doi: 10.5306/wjco.v13.i9.758. PMID: 36212602 PMC9537502

[12] Yu G Wang L-G Han Y He Q-Y . clusterProfiler: an R package for comparing biological themes among gene clusters. OMICS. (2012) 16:284–7. doi: 10.1089/omi.2011.0118. PMID: 22455463 PMC3339379

[13] Yu G . Using meshes for MeSH term enrichment and semantic analyses. Bioinformatics. (2018) 34:3766–7. doi: 10.1093/bioinformatics/bty410. PMID: 29790928

[14] Yoshimura K Suzuki Y Inoue Y Tsuchiya K Karayama M Iwashita Y . CD200 and CD200R1 are differentially expressed and have differential prognostic roles in non-small cell lung cancer. Oncoimmunology. (2020) 9. doi: 10.1080/2162402X.2020.1746554. PMID: 32395395 PMC7204521

[15] Chu K-H Chiang B-L . CD200R activation on naïve T cells by B cells induces suppressive activity of T cells via IL-24. Cell Mol Life Sci. (2024) 81:231. doi: 10.1007/s00018-024-05268-2. PMID: 38780647 PMC11116298

[16] Choueiry F Torok M Shakya R Agrawal K Deems A Benner B . CD200 promotes immunosuppression in the pancreatic tumor microenvironment. J Immunother Cancer. (2020) 8. doi: 10.1136/jitc-2019-000189. PMID: 32581043 PMC7312341

[17] Li J Wang Z Qin X Zhong MC Tang Z Qian J . CD200R1-CD200 checkpoint inhibits phagocytosis differently from SIRPα-CD47 to suppress tumor growth. Nat Commun. (2025) 16. doi: 10.1038/s41467-025-60456-3. PMID: 40461553 PMC12134331

[18] Kassiteridi C Cole JE Griseri T Falck-Hansen M Goddard ME Seneviratne AN . CD200 limits monopoiesis and monocyte recruitment in atherosclerosis. Circ Res. (2021) 129:280–95. doi: 10.1161/CIRCRESAHA.119.316062. PMID: 33975450 PMC8260471

[19] Valente T Serratosa J Perpiñá U Saura J Solà C . Alterations in CD200-CD200R1 system during EAE already manifest at presymptomatic stages. Front Cell Neurosci. (2017) 11:129. doi: 10.3389/fncel.2017.00129. PMID: 28522962 PMC5415594

[20] Snelgrove RJ Goulding J Didierlaurent AM Lyonga D Vekaria S Edwards L . A critical function for CD200 in lung immune homeostasis and the severity of influenza infection. Nat Immunol. (2008) 9:1074–83. doi: 10.1038/ni.1637. PMID: 18660812

[21] Kobayashi T Tanaka K Fujita T Umezawa H Amano H Yoshioka K . Bidirectional role of IL-6 signal in pathogenesis of lung fibrosis. Respir Res. (2015) 16. doi: 10.1186/s12931-015-0261-z. PMID: 26289430 PMC4546032

[22] Fielding CA Jones GW McLoughlin RM McLeod L Hammond VJ Uceda J . Interleukin-6 signaling drives fibrosis in unresolved inflammation. Immunity. (2014) 40:40–50. doi: 10.1016/j.immuni.2013.10.022. PMID: 24412616 PMC3919204

[23] O’Reilly S Ciechomska M Cant R van Laar JM . Interleukin-6 (IL-6) trans signaling drives a STAT3-dependent pathway that leads to hyperactive transforming growth factor-β (TGF-β) signaling promoting SMAD3 activation and fibrosis via Gremlin protein. J Biol Chem. (2014) 289:9952–60. doi: 10.1074/jbc.M113.545822. PMID: 24550394 PMC3975039

[24] Astrab LR Skelton ML Caliari SR . M2 macrophage co-culture overrides viscoelastic hydrogel mechanics to promote IL-6-dependent fibroblast activation. Cell Biomaterials. (2025) 1:100052. doi: 10.1016/j.celbio.2025.100052. PMID: 41798230 PMC12962745

[25] Schütz A Röser K Klitzsch J Lieder F Aberger F Gruber W . Lung adenocarcinomas and lung cancer cell lines show association of MMP-1 expression with STAT3 activation. Transl Oncol. (2015) 8:97–105. doi: 10.1016/j.tranon.2015.02.002. PMID: 25926075 PMC4415137

[26] Jiang YN Yan HQ Huang XB Wang YN Li Q Gao FG . Interleukin 6 trigged ataxia-telangiectasia mutated activation facilitates lung cancer metastasis via MMP-3/MMP-13 up-regulation. Oncotarget. (2015) 6:40719–33. doi: 10.18632/oncotarget.5825. PMID: 26528698 PMC4747364

[27] Kalluri R . The biology and function of fibroblasts in cancer. Nat Rev Cancer. (2016) 16:582–98. doi: 10.1038/nrc.2016.73. PMID: 27550820

[28] Yang H Jiang P Liu D Wang H-Q Deng Q Niu X . Matrix metalloproteinase 11 is a potential therapeutic target in lung adenocarcinoma. Mol Ther Oncolytics. (2019) 14:82–93. doi: 10.1016/j.omto.2019.03.012. PMID: 31024988 PMC6477516

[29] Zheng C-L Lu Q Zhang N Jing P-Y Zhang J-P Wang W-P . Comprehensive analysis of the immune and prognostic implication of MMP14 in lung cancer. Dis Markers. (2021) 2021:5917506. doi: 10.1155/2021/5917506. PMID: 34868395 PMC8635876

[30] Ye Q Taleb SJ Zhao J Zhao Y . Emerging role of BMPs/BMPR2 signaling pathway in treatment for pulmonary fibrosis. BioMed Pharmacother. (2024) 178:117178. doi: 10.1016/j.biopha.2024.117178. PMID: 39142248 PMC11364484

[31] Chung Y-H Huang Y-H Chu T-H Chen C-L Lin P-R Huang S-C . BMP-2 restoration aids in recovery from liver fibrosis by attenuating TGF-β1 signaling. Lab Invest. (2018) 98:999–1013. doi: 10.1038/s41374-018-0069-9. PMID: 29789683

[32] Arndt S Karrer S Hellerbrand C Bosserhoff AK . Bone morphogenetic protein-6 inhibits fibrogenesis in scleroderma offering treatment options for fibrotic skin disease. J Invest Dermatol. (2019) 139:1914–1924.e6. doi: 10.1016/j.jid.2019.02.020. PMID: 30878675

[33] Guan R Yuan L Li J Wang J Li Z Cai Z . Bone morphogenetic protein 4 inhibits pulmonary fibrosis by modulating cellular senescence and mitophagy in lung fibroblasts. Eur Respir J. (2022) 60:2102307. doi: 10.1183/13993003.02307-2021. PMID: 35777761 PMC9808813

[34] Ruzinova MB Schoer RA Gerald W Egan JE Pandolfi PP Rafii S . Effect of angiogenesis inhibition by Id loss and the contribution of bone-marrow-derived endothelial cells in spontaneous murine tumors. Cancer Cell. (2003) 4:277–89. doi: 10.1016/s1535-6108(03)00240-x. PMID: 14585355

[35] Mouillesseaux KP Wiley DS Saunders LM Wylie LA Kushner EJ Chong DC . Notch regulates BMP responsiveness and lateral branching in vessel networks via SMAD6. Nat Commun. (2016) 7:13247. doi: 10.1038/ncomms13247. PMID: 27834400 PMC5114582

[36] Jain RK . Normalization of tumor vasculature: an emerging concept in antiangiogenic therapy. Sci (1979). (2005) 307:58–62. doi: 10.1126/science.1104819. PMID: 15637262

[37] Qian C Liu C Liu W Zhou R Zhao L . Targeting vascular normalization: a promising strategy to improve immune-vascular crosstalk in cancer immunotherapy. Front Immunol. (2023) 14:1291530. doi: 10.3389/fimmu.2023.1291530. PMID: 38193080 PMC10773740

