# Supplementary figures and images for: Divergent immune and extracellular matrix transcriptional programs underlie poor prognosis in early-stage lung adenocarcinoma
Source: Front Immunol. 2026 Apr 16;17:1762224. doi: 10.3389/fimmu.2026.1762224 (PMC13128801; doi:10.3389/fimmu.2026.1762224)

# Distribution of GO–Term Hazard Ratios

Wilcoxon rank–sum  $p = 0.0185$

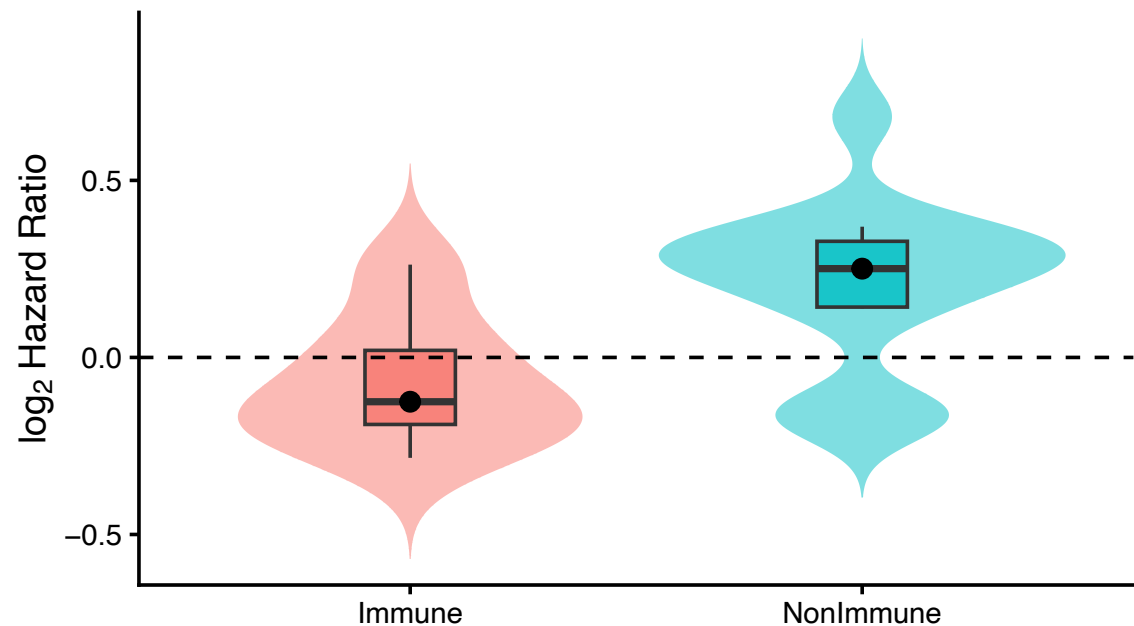

Supplement: Supplementary Figure S1 [file Image1.pdf]
